# In Individuals with Osteogenesis Imperfecta, Cephalometric Findings Suggest that Bisphosphonate Therapy May Improve Craniofacial Growth

**DOI:** 10.1007/s00223-025-01476-5

**Published:** 2026-01-31

**Authors:** Henri Tuurala, Janna Waltimo-Sirén, Helena Valta, Heidi Arponen

**Affiliations:** 1 Department of Pediatric Dentistry and Orthodontics, University of Turku, FI-20520 Turku, Finland; 2 Wellbeing Services County of South-West Finland, FI-20520 Turku, Finland; 3 Pediatric Research Centre, Children’s Hospital, University of Helsinki and Helsinki University Hospital, FI-00290 Helsinki, Finland; 4 Department of Oral and Maxillofacial Diseases, University of Helsinki and Helsinki University Hospital, FI-00290 Helsinki, Finland; 5 Department of Pediatric Dentistry and Orthodontics, Institute of Dentistry, University of Turku, Lemminkäisenkatu 2, FI-20520 Turku, Finland

**Keywords:** Osteogenesis imperfecta, Bisphosphonate therapy, Craniofacial development, Cephalometric analysis, Growth

## Abstract

Osteogenesis imperfecta is a rare hereditary condition affecting type 1 collagen formation. Among the wide spectrum of phenotypic features, osteogenesis imperfecta variably impairs craniofacial growth, affecting facial morphology and predisposing to malocclusion. At present, bisphosphonates are the gold standard for treatment of osteogenesis imperfecta, but knowledge on the effect of the medication on craniofacial growth is lacking. This retrospective study analysed lateral skull radiographs of 36 growing individuals with osteogenesis imperfecta and bisphosphonate treatment history (mean age 10.0 years, 13 females). Of them, 23 had been diagnosed with type I, 8 with type III, and 5 with type IV osteogenesis imperfecta. The historical control group that had not received bisphosphonate therapy comprised 34 individuals (mean age 8.1 years, 22 females) with osteogenesis imperfecta, type I in 18, type III in 7, and type IV in 9 individuals. The cephalometric measurement values were converted into age- and sex-matched Z-scores based on normal population values of the same ethnicity. Inter-group comparisons of the Z-scores showed several statistically significant differences where the bisphosphonate treatment group deviated less from unaffected population than the historical group. These included one or more of the mandibular size measurements in all studied types of osteogenesis imperfecta, anterior facial height and maxillary length in type IV, as well as cranial base angle, posterior facial height and angulation between the jaws in type III. Our findings suggest that bisphosphonate therapy may positively enhance both mid-facial and mandibular craniofacial growth in individuals with osteogenesis imperfecta.

## Introduction

Osteogenesis imperfecta (OI) is a rare, hereditary connective tissue disorder, where usually the formation, quantity, and/or structure of type 1 collagen is aberrant. The most common cause of OI is an autosomal dominant pathogenic variant in either *COL1A1* or *COL1A2* gene, which encode amino acid chains of type 1 collagen. Although most of these pathogenic variants affect directly type 1 collagen synthesis, pathogenic variants have been found affecting other aspects of osteoblast function, some of which have autosomal recessive or X-linked recessive heritability [1, 2]. Common features present among individuals with OI include recurring bone fractures, increased flexibility of joints and skin, hearing loss, and - in severe disorder forms - short stature. Additionally, depending on the type of the disease and the severity of the symptoms, individuals with OI may experience scoliosis, cranial base deformity, sleep apnea, malocclusion, developmental abnormality of dentin, and blue sclerae [2–6]. Currently, OI has been identified as having 22 genetically determined subclasses [7]. The Nosology Group of the International Society of Skeletal Dysplasias has recommended that due to the diverse genotypic nature of OI, the classification of disease severity should use the original, simpler Sillence classification I-IV with addition of OI type V [8, 9], type V showing distinct clinical and radiological features and genetic background [10]. The prevalence of all different types of OI worldwide in the population is approximately 1:10,000–20,000 [11].

In line with the general severity of OI, among those surviving beyond early childhood, facial clinical and lateral cephalometric features differ from general population most in types III and IV OI and least in type I [12–15]. A similar difference between OI types has been reported for the frequency and severity of malocclusion in the mixed and permanent dentition [16, 17]. Common forms of malocclusion include anterior and posterior cross bites and open bites. Adolescents with OI and posterior malocclusion, in particular, have reported functional limitation that has a negative impact on oral health-related quality of life [18].

Frequently, a deviant anterior-posterior relationship of the jaws -relative mandibular prognathism- often manifests as class III molar relationship and both anterior and posterior cross-bites. Cephalometric analysis of lateral skull radiographs has revealed that in OI, the characteristic sagittal jaw relationship results from a hypodivergent growth pattern caused by restricted vertical development of the jaw and, particularly, alveolar bone [13]. The development of open bite in this patient group in turn may stem from a mismatch between the normal-sized tongue and the restricted oral cavity space, and thereby an altered resting tongue position between or against the dental arches [19].

Although there is no cure for OI, bisphosphonates, as anti-resorptive drugs that inhibit osteoclast activity and thereby improve bone mineral density, currently form the gold standard in the medical treatment of OI. However, recent advancements in medication therapy indicate a growing focus on the development and use of novel drugs and treatment modalities [20, 21]. Despite the widespread use of bisphosphonates to alleviate clinical symptoms of OI, the assessment of craniofacial features, underlying the frequent and severe malocclusions in patients with OI, originates from an era before bisphosphonate treatment. Additionally, previously published assessments do not allow for a comparison between treated and untreated patients. The aim of the present study is to analyse the impact of bisphosphonate therapy on craniofacial size and form, the therapy been ongoing during growth in individuals with OI.

## Materials and Methods

### Study Material

This cross-sectional retrospective study analysed lateral skull radiographs of young, growing individuals with OI, comparing the craniofacial form and dimensions of those who received bisphosphonate therapy during growth with those who had not received bisphosphonates. Cut-off age was set at 20 years, up to an age where minute craniofacial growth changes are still ongoing [22].

The study population was representative of the target population and comprised all growing individuals with OI who had a history of bisphosphonate therapy and were treated in Helsinki University Hospital. OI was determined through genetic testing. The inclusion criteria were pathogenic variant of *COL1A1* or *COL1A2* gene leading to type I OI (the mild type), type IV OI (moderate in severity), or type III OI (the severe type) by Sillence classification [8], a lateral skull radiograph of diagnostic quality available, obtained between the ages 2 to 20 years, and bisphosphonate therapy started a minimum of 1 year before obtaining the radiograph. In total, 46 individuals were excluded due to missing radiographs. For a few patients several lateral skull radiographs were available, but only one radiograph of good quality per each individual was included. All radiographs had been taken prior to the study for valid diagnostic reasons.

The patients of our cohort had been on pamidronate or zoledronic acid treatment. Pamidronate was started for the subjects under the age of two years and then switched to zoledronic acid. Pamidronate was given intravenously for three consecutive days with the dose of 0.5 mg/kg every second month for children under two years and 0.75 mg/kg every third month for those between two and three years of age. Zoledronic acid infusions were administered 0.025–0.05 mg/kg up to 4.0 mg/day once every 6 months. Bisphosphonate treatment was continued throughout the child’s growth period.

All the patients were on vitamin D supplementation and the target plasma level of vitamin D was 75–120 nmol/L. The doses of calcium supplementation varied between 250 mg to 1000 mg/day depending on the age of the child and the use of dairy products. During bisphosphonate infusions the calcium doses were doubled for 2 weeks.

All radiographic measurements were compared with values from a longitudinal growth study [22]. It comprises detailed craniofacial growth data on Finns from 4 to 25 years, based on series of altogether 551 lateral cephalograms of 105 unselected and orthodontically non-treated individuals. The individuals in the longitudinal growth study had a geographically wide-spread background among the Finnish population and represented the same ethnicity as the study and control groups.

Based on medical records, no fractures in the craniofacial bones had occurred. The patients’ heights were obtained from medical records within a maximum of three-month range from the time point of obtaining the radiograph. Height measurement was available for 61 individuals. Height Z-value was calculated using data from the longitudinal growth study [22].

### Measurements

**Fig. 1:**
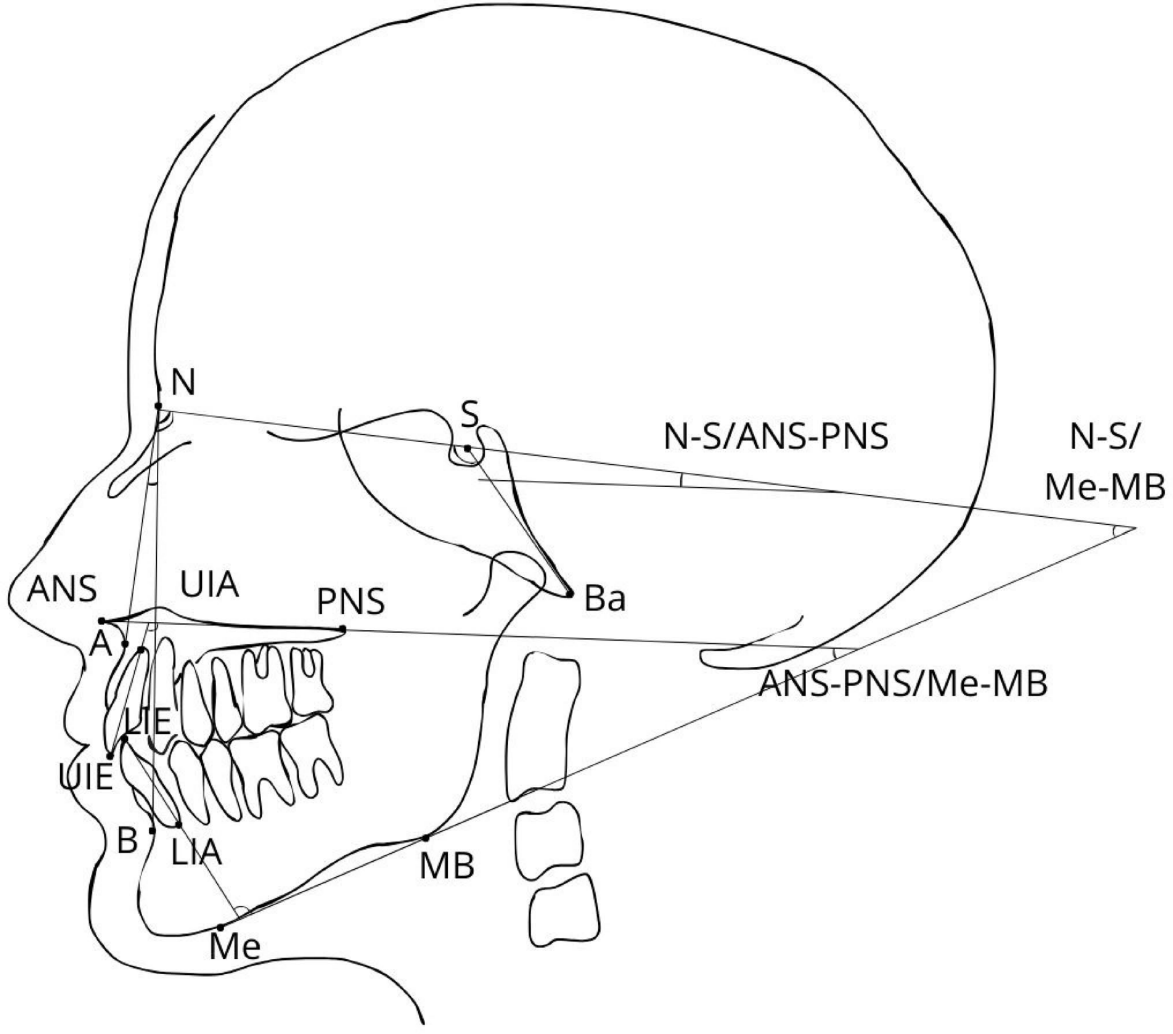
The reference points and lines used for angular measurements in the cephalometric analysis in alphabetic order. A, A-point; ANS, anterior nasal spine; B, B-point; Ba, basion; LIA, lower incisor apex; LIE, lower incisor edge; MB, mandibular base; Me, menton; N, nasion; PNS, posterior nasal spine; S, sella; UIA, upper incisor apex; UIE, upper incisor edge. Explanations for depicted angular measurements: Angulation between the cranial base and palatal plane (N-S / ANS-PNS), Angulation between the jaws (ANS-PNS / Me-MB), Cranial base angle (NSBa), Facial angle (N-S / Me-MB), Inclination of lower incisors to mandibular plane (LIE-LIA / Me-MB), Inclination of upper incisors to palatal plane (UIE-UIA / ANS-PNS), Mandibular sagittal position (SNB), Maxillary sagittal position relative to the cranial base (SNA), Sagittal relationship between the jaws (ANB)

**Fig. 2:**
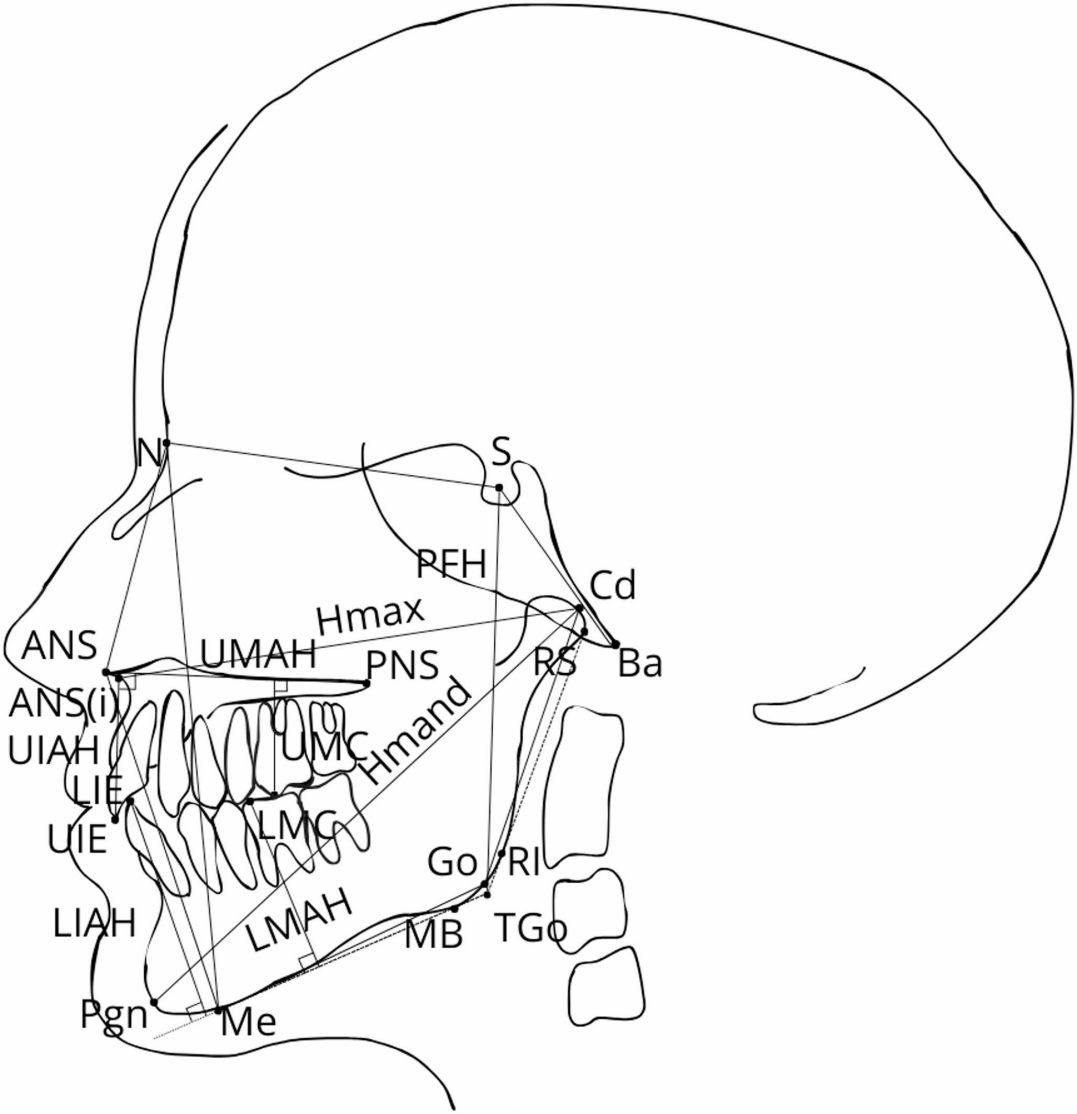
The reference points used for linear measurements in alphabetic order: ANS, anterior nasal spine; ANS (i), a point on the lower contour of the anterior nasal spine where the vertical thickness is 3 mm; Ba, basion; Cd, condylion; LIE, lower incisor edge; LMC, lower molar cusp; MB, mandibular base; Me, menton; N, nasion; Pgn, prognathion; PNS, posterior nasal spine; RI, ramus inferior; RS, ramus superior; S, sella; TGo, constructed gonion; UIE, upper incisor edge; UMC, upper molar cusp. Explanations for depicted linear measurements: Hmand, mandibular unit length as defined by Harvold (Pgn-Cd); Hmax, maxillary unit length as defined by Harvold (ANS(i)-Cd); LIAH, lower incisor alveolar height (LIE perpendicular to Me-MB), LMAH, lower molar alveolar height (LMC perpendicular to Me-MB); PFH, posterior facial height (S-TGo); UIAH, upper incisor alveolar height (UIE perpendicular to ANS-PNS); UMAH, upper molar alveolar height (UMC perpendicular to ANS-PNS)

Cephalometric points, angular and linear measurements are illustrated in Figs. 1 and 2. The cephalometric landmarks were identified by an orthodontist (H.A.) and a general dentist ( H.T.) until consensus was reached. The lateral skull film radiographs were analysed manually and the digital ones using Viewbox software (version 4.0.1.7, dHAL Software). Previous studies have shown that the two methods are comparable in reliability [23]. The length of the mandible and the effective length of maxilla were measured according to Harvold [24]. For linear measurements, magnifications were corrected to natural size. Each measurement was subsequently converted into age- and sex-matched relative Z-scores based on the normal population values.

### Reliability Analysis

Landmark identification and cephalometric measurements were re-done on 22 randomly picked radiographs by the same two examiners independently to assess for intra-examiner random errors using the Dahlberg formula [25].

### Statistical Analysis

In the statistical analysis, OI types I, III, and IV were analysed as separate groups. The statistical analyses were performed with the software programs JMP Pro (version 17.0.0, SAS Institute Inc.) and SPSS statistics (version 27, IBM). Shapiro-Wilk Test of normality was applied to examine the distribution of the data. The characteristics of the study groups were compared with Fisher’s exact test and a chi-squared test. The association between the age at the onset of bisphosphonate treatment and OI type was examined with Spearman’s rank correlation test. Mann-Whitney U-test was applied to assess differences in age and measurement Z-scores between the groups; patients with bisphosphonate therapy history and those without. All available data were included in the analysis, and missing data were not imputed. Statistical significance was set at 0.05 level.

## Results

The average intra-examiner random measurement error was 1–2 degrees for the angular measurements and 1 mm for the linear measurements. We therefore consider the method reliable [25, 26].

The final study group of 36 individuals represented OI types I (23 individuals), III (8 individuals), and IV (5 individuals). Their age range was from 3.9 to 18.1 years, and the mean age was 10.0 years (Table 1). Of the participants, 23 were males and 13 were females.

**Table 1:** Description of the two groups of patients with osteogenesis imperfecta (OI), a contemporary group that has been on bisphosphonate treatment and a historical group without bisphosphonate treatment

	Type I OI		Type III OI		Type IV OI		All OI types	
Bisphosphonate treatment	Yes	No	Yes	No	Yes	No	Yes	No
N	23	18	8	7	5	9	36	34
Females (%)	35	61	50	86	20	56	36*^a^	65
*Age*								
Mean (yrs)	11.0*^b^	7.6	6.7	11.1	11.4*^b^	6.9	10.0*^b^	8.1
Range (yrs)	5.6–18.1	3.8–16.8	3.9–15.1	3.5–15.1	9.2–14.0	6.0–8.2	3.9–18.1	3.5–16.8
SD (yrs)	3.4	3.4	2.3	4.2	2.3	0.8	3.6	3.4
*Height Z-score*								
Mean	−1.0	−1.1	−5.4*^b^	−7.0	0.1*^b^	−4.3	−1.9	−2.7
Range	−2.3–0.5	−7.0–1.4	−8.0–−2,7	−7.0–−7.0	−1.7–2.6	−6–−1.6	−8.0–2.6	−7.0–1.4
*Time from the onset of bisphosphonate treatment*								
Mean (yrs)	6.1		5.5		4.2		5.7	
SD (yrs)	4.1		2.1		2.5		3.5	

*p < 0.05; ^a^Fisher’s exact test; ^b^Mann-Whitney U-test

The control group consisted of lateral cephalograms collected from 34 growing individuals with OI, representing OI types I (18 individuals), III (7 individuals), and IV (9 individuals), by clinical phenotyping. This group of patients comprised 12 males and 22 females, and their ages ranged between 3.5 and 16.8 years, with the mean age being 8.1 years. The craniofacial findings of this patient sample have been previously published [13], but the radiographs were re-evaluated for the present study.

The subgrouping of OI according to the clinical severity of the disease among the 36 patients who had been on bisphosphonate therapy (BT group) and the 34 who had not been treated (NT group), are shown in Table 1. Considering the whole study population, there were statistically significantly less women in the BT group than in the NT group (*p* = 0.017), but within the three clinical OI subgroups, differences between the numbers of females and males did not reach statistical significance (Fisher’s exact test). The three clinical subtypes of OI were not statistically significantly different in terms of the distribution of NT and BT groups (chi-squared test, χ^2^ = 1.75, *df* = 2, *p* = 0.42).

The individuals in the BT group were statistically significantly older at the time of imaging than were those in the NT group (U = 393.5, *p* = 0.010) when considering the whole cohort. Group-specifically, among those with type I OI, the ones with bisphosphonate therapy were older than the historical controls at the time of radiography (U = 321.5, *p* = 0.003). An identical difference was noted among those with type IV OI (U = 45.0, *p* = 0.001). In contrast, the distribution of age did not differ across the groups of individuals with type III OI (U = 11.0, *p* = 0.054).

In the BT group, the bisphosphonate treatment was started earliest at the age of 0.1 years, and latest at the age of 13.2 years. The mean age at the onset of bisphosphonate treatment was 4.2 years (SD 3.6 years). The therapy was started earlier in individuals with severe OI type (r_s_= -0.48, *p* = 0.003, *n* = 36) suggesting clinical indication for the bisphosphonate therapy, and probably an earlier diagnosis in cases with more severe OI types. Minimum and maximum time lapses between the onset of bisphosphonate treatment and the radiography were 1.0 year and 15.0 years, mean 5.7 years (SD 3.5 years) (Table 1).

The distribution of height Z-scores was similar across the study and control groups in the whole cohort (U = 389.0, *p* = 0.309, *n* = 61) and among those with OI type I (U = 143.0, *p* = 0.511, *n* = 37) (Table 1). In contrast, individuals with types III or IV OI in the BT group were taller than were those in the NT group (U = 30.5, *p* = 0.008, *n* = 12 and U = 27.0, *p* = 0.012, *n* = 11, respectively).

Regarding facial size and form (Table 2), the number of study subjects varied between the different measurements within each group, as some radiographs were lacking a scale, had an incomplete field of view, or the patient’s positioning was compromised.

**Table 2:** Craniofacial measurements of patients with osteogenesis imperfecta (OI), and with or without a history of bisphosphonate treatment

Bisphosphonate therapy	Definition	Type I OI						Type III OI						Type IV OI					
		Yes (n=23)			No (n=18)			Yes (n=8)			No (n=7)			Yes (n=5)			No (n=9)		
		n	Mean	SD	n	Mean	SD	n	Mean	SD	n	Mean	SD	n	Mean	SD	n	Mean	SD
* Cranial base measurements (Z-scores)*																			
Anterior cranial base length	N-S	22	−0.56	1.61	18	−0.72	2.00	5	−0.61	2.22	7	−1.59	1.51	3	−0.48	1.06	9	−1.41	1.75
Clivus length	S-Ba	22	−0.26	1.01	18	0.05	1.83	5	−1.57	2.34	7	−1.78	1.85	3	−0.97	0.62	9	−0.98	1.51
Cranial base angle	N-S-Ba	23	1.76	1.12	18	0.98	1.25	**8**	4.76*	**2.76**	**7**	**8.02**	**3.53**	5	2.37	1.86	9	2.79	1.34
*Facial measurements (Z-scores)*																			
Facial angle	N-S / Me-MB	19	1.36	1.21	18	0.75	1.37	4	0.38	0.74	7	2.17	2.29	4	1.17	1.08	9	1.08	0.98
Anterior facial height	N-Me	19	0.61	1.31	18	0.46	1.65	4	−1.57	2.31	7	−2.31	1.39	**2**	1.41*	**0.10**	**9**	**−0.46**	**0.66**
Upper anterior facial height	N-ANS	22	0.10	1.06	18	0.34	1.41	4	−0.27	1.13	7	−0.85	1.27	2	−1.04	2.56	9	0.05	0.91
Lower anterior facial height	ANS-Me	19	0.65	1.38	18	0.77	1.46	4	−1.87	1.95	7	−1.66	1.17	**2**	2.11*	**1.46**	**9**	**−0.15**	**0.31**
Relative height of the lower anterior face (%)	ANS-Me / N-Me	19	0.44	1.31	18	0.81	1.32	4	−1.73	1.10	7	−0.44	1.25	4	1.04	2.17	9	0.30	0.79
Posterior facial height	S-TGo	19	−0.36	0.97	18	0.04	1.77	**4**	−1.99*	**1.13**	**7**	**−4.27**	**1.64**	2	0.21	0.70	9	−0.73	0.75
Relation of posterior and anterior facial heights (%)	S-TGo / N-Me	19	−0.86	0.89	18	−0.33	1.47	4	−0.83	0.62	7	−2.57	1.98	4	−0.81	0.98	9	−0.31	0.98
*Maxillary and mandibular measurements (Z-scores)*																			
Inclination of maxilla in relation to cranial base	N-S / ANS-PNS	23	1.17	1.22	18	0.62	1.19	8	3.46	0.72	7	3.31	2.03	5	1.88	2.34	9	1.61	1.35
Angulation between the jaws	ANS-PNS / Me -MB	19	0.80	1.38	18	0.41	1.38	**4**	−2.41*	**0.29**	**7**	**0.16**	**1.52**	4	0.39	1.57	9	0.09	0.79
Length of the maxilla	ANS-PNS	22	0.68	1.61	18	0.31	1.36	5	0.34	1.86	7	−0.51	0.78	**3**	1.68*	**1.24**	**9**	**−0.45**	**1.03**
Length of the mandibular ramus	Cd-Go	**21**	0.22*	**1.33**	**18**	**−0.70**	**1.48**	**5**	−0.97*	**1.21**	**7**	**−4.73**	**1.84**	2	0.39	1.86	9	−1.67	1.30
Length of the mandibular corpus	Me-Go	**21**	−1.63*	**1.51**	**18**	**−0.58**	**1.74**	5	−2.48	1.95	7	−1.94	1.53	2	−0.77	0.63	9	−1.92	1.14
Sagittal position of the maxilla	S-N-A	23	−0.40	0.92	18	−0.39	1.71	8	−2.05	0.78	7	−3.45	2.00	5	−0.68	1.72	9	−0.54	1.20
Sagittal position of the mandible	S-N-B	19	−0.40	1.16	18	−0.41	2.04	4	−1.45	1.68	7	−3.28	2.74	4	−1.19	1.68	9	−0.75	0.85
Sagittal relationship between the jaws	A-N-B	19	0.07	1.47	18	0.19	1.44	4	−1.08	2.15	7	−0.42	1.57	4	0.99	1.28	9	0.25	1.64
Length of the maxilla by Harvold (Hmax)	Cd-ANS(i)	21	1.23	1.56	18	0.58	1.59	5	−0.62	1.45	7	−2.14	1.03	2	0.68	1.80	9	−1.01	0.79
Length of the mandible by Harvold (Hmand)	Cd-Pgn	21	−0.04	1.40	18	−0.46	2.11	5	−2.75	1.36	7	−4.48	1.86	**2**	0.41*	**0.01**	**9**	**−2.07**	**1.32**
Harvold difference	Hmand - Hmax	21	−1.09	1.00	18	−1.25	1.39	5	−2.63	1.21	7	−3.41	2.27	2	−0.10	1.46	9	−1.32	1.29
*Dentoalveolar measurements (Z-scores)*																			
Inclination of upper incisors to palatal plane	UI / ANS - PNS	22	0.57	1.48	18	0.13	1.71	7	2.83	2.14	6	1.82	2.37	5	0.92	1.47	7	1.11	1.84
Inclination of lower incisors to mandibular plane	LI / Me – MB	22	−0.72	1.03	18	−0.26	1.07	6	1.30	1.64	7	0.26	1.97	5	0.02	1.36	8	−0.33	1.10
Alveolar height of upper incisors	UIE to ANS-PNS	21	−0.64	1.49	16	−0.15	1.59	5	−2.92	2.02	6	−2.93	1.78	3	0.38	2.33	5	−0.77	0.69
Alveolar height of upper first molar	UMC to ANS-PNS	22	−0.41	0.88	18	0.45	1.75	5	−2.46	1.67	7	−2.84	1.10	3	−0.54	2.99	8	−0.17	1.27
Alveolar height of lower incisors	LIE to Me-MB	22	0.44	1.53	17	0.25	2.19	5	−1.67	0.77	7	−2.65	1.45	3	0.88	1.25	5	0.32	1.44
Alveolar height of lower first molar	LMC to Me-MB	22	−0.43	0.94	18	0.21	1.60	5	0.02	2.62	6	−2.29	1.06	3	−0.75	1.57	8	−0.80	1.58

* *p* < 0.05. All measures are expressed as Z-scores in comparison to healthy population values. The bold figures refer to measurements with statistically significantly different Z-scores between the groups with and without medication within each clinical type of OI

In individuals with OI type I, all facial measurements by mean were within a ± 2.0 Z-score range from population mean norms. The most extensive variation from population mean was observed in the cranial base angle that was larger in individuals with OI (Z-score 1.76, BT group). Comparison between the BT and NT groups revealed a statistically significant difference in mandibular ramus length (Cd-Go, *n* = 39, U = 104.0, *p* = 0.016) and mandibular corpus length (Me-Go, *n* = 39, U = 261.0, *p* = 0.043) Z-scores, indicating a possibly improved vertical growth of the articulating part of the mandible in the BT group (Table 2). Interestingly, the mandibular corpus seemed to be shorter in the BT group, leading to the total mandible length being similar between the BT and NT groups.

In individuals with type IV OI, among the different measurements, the cranial base angle differed most from the normative values, displaying Z-scores 2.37 in BT and 2.79 in NT groups, suggestive of platybasia. Other Z-score means exceeding ± 2.0 were lower anterior facial height (Z-score 2.11, BT group) and length of the mandible by Harvold (Z-score − 2.07, NT group). The inter-group difference between the BT and NT groups in the mandibular ramus length was even more extensive in those with OI type IV compared to those with type I, indicative of enhanced growth in the treated individuals, with a mean Z-score difference of 2.06. However, the difference did not reach statistical significance, probably due to small group sizes. Statistically significant differences were observed in anterior facial height (N-Me, *n* = 11, U = 0.000, *p* = 0.036), lower anterior facial height (ANS-Me, *n* = 11, U = 0.000, *p* = 0.036), maxillary length (ANS-PNS, *n* = 12, U = 1.00, *p* = 0.018), and the entire mandible length (Cd-Pgn, *n* = 11, U = 0.000, *p* = 0.036). All of these measurements were larger in the BT group, indicating growth improvement not only vertically but also in the anterior-posterior dimension.

In the type III OI group, some of the linear measurement Z-scores were considerably low, whereas several angular measurement Z-scores were high, which indicates a craniofacial morphology that by mean markedly differs from one of age- and sex-matched healthy population controls. In the historical NT group, mean Z-scores were indicative of platybasia (cranial base angle; 8.02), short total mandibular length (from the condylar head to the chin prominence; −4.48) that was mostly due to deficient growth of the ascending part of the mandible (from the condylar head to gonial angle; −4.73). Vertical growth deficiency was also expressed as low posterior facial height (measured from sella to constructed gonion; −4.27) and low alveolar heights (from −2.93 to −2.29). Angles differing most from normative ones list the angulation of the palatal plane in relation to anterior skull base (3.31), and SNA and SNB angles (−3.45 and −3.28, respectively) indicative of both maxillary and mandibular retrognathia. Notably, a low position of the sella affects all these angular measurements.

Among patients with type III OI, in the BT group, cranial base angle (mean Z-score 4.76 in BT), and mandibular ramus length (−0.97 in BT) were closer to population norms with a Z-score difference between BT and NT exceeding as much as 3.0. A Z-score difference of a minimum of 2.0 was observed regarding posterior facial height (−1.99 in BT), angulation between the jaws (−2.41 in BT), and alveolar height of the lower first molar (0.02 in BT). Of the inter-group differences the following were statistically significant: cranial base angle (N-S-Ba, *n* = 15, U = 46.0, *p* = 0.040), posterior facial height (S-TGo, *n* = 11, U = 3.00, *p* = 0.042), angulation between the jaws (ANS-PNS / Me-MB, *n* = 11, U = 28.0, *p* = 0.006), and mandibular ramus length (Cd-Go, *n* = 12, U = 1.00, *p* = 0.005). These linear measurements were larger and angular measurements smaller in the BT group than in the NT group, bringing the variables closer to population norms.

Subgroup analyses by sex revealed a statistically significant difference in females with OI type III between NT and BT groups regarding the angle between jaws (ANS-PNS / Me-MB, *n* = 9, U = 0.00, *p* = 0.024) and mandibular ramus length (Cd-Go, *n* = 9, U = 0.00, *p* = 0.24), with the angle between jaws being smaller in BT group and mandibular ramus length being larger in BT group. In males with type I OI, statistically significant difference was observed in mandibular corpus length (Go-Me, *n* = 19, U = 12.0, *p* = 0.017), it being smaller in the BT group.

## Discussion

This study examined craniofacial dimensions in growing individuals with OI and eventual effects of bisphosphonate treatment on craniofacial complex. We found that the craniofacial dimensions of individuals with bisphosphonate therapy received in childhood differ from those of individuals with no therapy, the former being closer to unaffected population, which implies that bisphosphonates affect craniofacial growth positively if any. To the best of our knowledge, this is the first study to report the effects of bisphosphonate treatment on craniofacial structures in humans.

Similar to the spine and long bones, the bones of the cranial base form through endochondral ossification of primary cartilage. Cartilage bands, known as synchondroses, that histologically resemble two-sided epiphyseal plates remain between the cranial base bones. These are sites of active cartilage cell proliferation and endochondral growth until they gradually close from birth to adolescence, leading to the fusion of individual bones [27, 28]. Platybasia, a flatter than normal cranial base, is frequently seen in patients with OI, and detected from birth [29]. We suggest that platybasia develops in the fetus due to delayed ossification of the cranial base in relation to normal expansive growth of the neural tissue, or else, as abnormal response to the compressive and tensile stresses on the already formed bone that induce bone resorption on the endocranial surface and bone formation ectocranially [30]. The previous explanation is in line with the findings in calvaria with a large anterior fontanelle at birth in newborns with the perinatal lethal type II OI, and prenatal formation of numerous extra bones, Wormian bones, in the sutural areas [31]. These can be interpreted to indicate that the flat, intramembranously forming calvarial bones that protect the brain dorsally grow too slowly in response to the brain expansion as well. The calvaria expands as a response to growth of the brain by sutural growth and by resorption from ventral surfaces along with apposition dorsally [32]. If bisphosphonate treatment starts early, the surface modelling might be affected, including sutures, where osteoclastic activity plays a role in the maintenance of suture patency [32].

In the present study, the cranial base anterior to sella was by mean shorter than population mean in all OI types studied, and in both NT and BT groups, but closer to normal in each OI type after bisphosphonate treatment. It was the shortest in untreated individuals with type III OI. Clivus length was at population norms in type I OI, shorter in type IV, and shortest in type III, and without any suggestive effect of bisphosphonates. While the linear measurements all remained within two-Z-score range from normal, there were markedly aberrant scores for the cranial base angle, which was larger than normal in all OI-types and with a worsening gradient from type I to type IV and to type III, with a Z-score of 8.0 in the latter in patients not treated with bisphosphonates. While less pronounced flattening was noted in the BT groups compared to NT groups in type IV and particularly in type III, an opposite difference was seen in type I OI. Statistical significance was reached in OI type III, indicating that bisphosphonate therapy may delay the development of platybasia in severe form of OI.

In a large group of OI patients that were of the same age range as in the present study and nearly all being on bisphosphonate therapy, platybasia was observed in 62%, but notably 43% of the patients had OI type III [33]. It is thus evident that platybasia may develop, or more likely remain, despite bisphosphonate treatment. Whereas the clinical significance of platybasia as a solitary feature can be questioned, the orientation of the skull base affects all cephalometric measurements using sella-nasion line as a reference plane.

The growth of the nasomaxillary complex occurs by a passive and an active mechanism. Passive growth happens when the cranial base grows in size and pushes the maxilla forward. This growth slows down considerably at around the age of 7 years, when the spheno-ethmoidal synchondrosis closes. Active growth mechanisms of the facial bones, including maxilla that develop intramembranously without a cartilage model, comprise growth in the sutures between the individual bones and surface apposition and remodelling where osteoclasts also play a role. As the maxilla grows in size and volume, bone is resorbed from its anterior surface, partially counteracting its translation forwards [27, 28]. Resorption is critical in the lowering of the nasal floor and formation of the paranasal sinuses.

In this study, the length of the maxilla from anterior to posterior nasal spines was larger in all OI types in the BT group, and regarding type IV OI, the difference was statistically significant. This might reflect reduced resorption of the anterior maxilla. SNA-angles were below population norms in all OI types and both in NT and BT groups. In type III OI, the maxilla, based on SNA values, was less retrognathic in the treated patients than in the untreated ones. The anterior-posterior depth of the face, measured from the anterior nasal spine to the mandibular condyle, was in the BT groups larger than in the NT groups regarding all OI types, in type I OI by 0.65 Z-score, in type IV by 1.7, and in type III by 1.5, although none of these differences reached statistical significance.

The growth of the mandible occurs both intramembranously and endochondrally. Whereas the body of the mandible grows through appositional bone deposition and surface remodelling, a secondary cartilage forms in the articulating part, the condyle, and forms a site of endochondral growth, displaying cellular arrangement common with epiphyseal growth plates [28]. As the rest of the craniofacial structures and the cervical spine grow, the mandible translates forwards and downwards. As a response to this translation, the mandible grows upwards and backwards to maintain contact with the articulating fossa in the skull, whereas only very little growth happens in the chin area [28, 34]. As in maxillary growth, bone resorption takes place in the mandible as well, namely on the anterior surface of ramus and the area just above mental protuberance (B-point) [27, 28].

Here the total maximum length of the mandible, measured from the chin to condyle, was shorter than normal in all OI types in the NT groups, with the following Z-scores: −0.5 (type I), −2.1 (type IV) and −4.5 (type III). Findings in the groups treated with bisphosphonates were suggestive of a positive effect on mandibular growth in all OI types: in types I and III OI with intergroup differences of 0.4 and 1.7 Z-scores, respectively, while in type IV OI it was 2.5, which was a statistically significant difference. This was notably due to growth in the ramus, the vertical and articulating part of the mandible, where the respective intergroup differences, positive for bisphosphonate treatment, were 2.1 in type IV OI, and statistically significantly 0.9 in type I and 3.8 in type III.

A number of earlier mice and human studies support our observation of positive effect of bisphosphonates on mandibular condylar growth. Alendronate treatment on OI mice has been shown to slow down loading-induced degenerative effects in the temporomandibular joint (TMJ), helping to preserve the extracellular matrix volume in the subchondral bone and the cartilage [35]. In severe form OI mice, zoledronic acid has been found to increase the length and the mineral density of the mandibular ramus [36]. Additionally, cyclic pamidronate has been noticed to alter the trabecular bone architecture of the mandibular condyles in individuals with moderate and severe forms of OI [37]. It has also been documented to increase the mandibular cortical width in all OI types, more so in types I and IV [38].

Growth increment in the vertical height of the anterior face, measured from naso-frontal suture to the lower-most point of the jaw, is a combined result of sutural growth in the midface, mandibular growth and displacement of the glenoid fossa, and periosteal growth of the alveolar ridges [39]. Here, the differences in vertical facial height were suggestive of a positive effect of the medication. While the difference remained modest in OI types I and III with Z-scores of 0.2 and 0.7 respectively, the anterior face in OI type IV was statistically significantly higher in the BT group with a 1.9 Z-score difference. The latter was explained by improved growth not in the upper but in the lower part of the anterior face, measured down from the nasal spine, showing likewise a statistically significant Z-score difference of 2.6 in favour of the BT group in type IV OI. This finding would imply that the medication could benefit more endochondral and alveolar than sutural growth.

Posterior facial height, measured from the center of the pituitary fossa to a constructed gonion point, differed between the untreated and treated individuals, indicating a positive effect on growth of the bisphosphonate medication, in OI types III (Z-score difference 2.3, *p* = 0.042) and IV (Z-score difference 0.9). These differences are compatible with lowering of the mandibular angle, both due to the higher mandibular rami and more normal angulation of the cranial base, which likely contributes to a relative anterior and inferior displacement of the temporomandibular fossa, and more dorsal position of the pituitary fossa.

The dentoalveolar bone in both jaws was documented previously to be lower in height in a group of individuals comprising either type III or type IV OI, even explaining the hypodivergent growth pattern of the jaws and relative mandibular prognathism [13]. In the present study, types III and IV OI were analysed separately and the paired controls replaced by population norms. In the non-treated groups of patients (being same individuals as in aforementioned paper) the findings were that alveolar heights in type III OI were by 2.3 to 2.9 Z-scores lower than norms, and in type IV OI, somewhat lower than norms except for the lower incisors. Comparison of these with the alveolar heights of patients that had been on bisphosphonate therapy, gave inconsistent results. There are many plausible explanations for this. The alveolar bone is a reactive bony structure – its overall development and maintenance is dependent on the eruption and presence of teeth and thus of the dental developmental stage and dental health, and there is a negative correlation between its height and occlusal forces.

High dose zoledronic acid has been observed to inhibit tooth eruption in healthy mice, thus compromising alveolar bone height gain [40]. With therapeutic doses in children with OI and treated with bisphosphonates, the dental development has been shown to be relatively slowed down [41].

Skeletal class is a description of the harmony between maxillary and mandibular bones, class I being harmonious. Class II can be defined as disharmony, where the mandible relative to the maxilla is disproportionally small or retrognathic, and class III the opposite. Spatial sagittal relationship is best analysed through the ANB-angle, and size proportions through difference in the jaw sizes, e.g. utilizing the Harvold measurements. In healthy individuals, those with skeletal class III have a tendency to display class III molar relationship and anterior cross-bite.

The mean ANB-angles of the historical controls as well of those in the BT group were all within 1.1 Z-score distance from population norms. Hence, these mean values were suggestive of skeletal class I, but the ones with the smallest angulation and thus a tendency towards straight or concave profile were the representatives of the BT group among type III OI. Harvold jaw-size difference was at or below zero Z-scores for all groups, and it was the smallest and thus suggestive, on the contrary, of skeletal class II in the untreated individuals with type III OI. Thereby none of the groups at mean level showed any indication of class III skeletal relationship according to this measure. There thus seems to be a clear discrepancy between the well-documented increased frequency of class III malocclusion, particularly in patients with types III and IV OI [42] and the skeletal structure observed here on a group level, using common tools for skeletal class assessment. This emphasizes the unique characteristics of the facial proportions particularly in the severe type III OI, in line with an earlier report by Waltimo-Sirén et al. [13].

In a harmonious face, four planes, the skull base plane, the palatal plane, the occlusal plane, and the mandibular plane have been proposed to meet in one point situated posterior to the occipital contour [43]. Although this is very theoretical, it helps to understand disproportionate growth and growth rotations, where in a hypodivergent face, the planes are quite parallel and the contacting point moves far back, and in a hyperdivergent face there are large angles between the planes and they meet each other rather anteriorly.

A strongly closing, hypodivergent, growth rotation of the mandible has been described typical for OI type III, the statement based on the small angle between palatal and mandibular planes [13]. Here the angle was somewhat surprisingly, quite close to population mean in the untreated groups. In the type III BT group closing rotation occurred, with intergroup Z-score difference of −2.6 (*p* = 0.006). This implies that with improved growth of the mandibular ramus, described earlier, the mandible rotates anteriorly, which is in line with growth patterns in unaffected individuals. In types I and IV, no clear differences were shown between NT and BT groups. Facial angle, the angle between the skull base and mandibular planes, as shown before [13], is a poor indicator of growth rotation in OI, and the same stands true for the relationship between posterior and anterior facial heights, where the position of the pituitary fossa in relation to other craniofacial structures interferes with the interpretation.

Signs suggestive of positive effects from the treatment with bisphosphonates were observed here regarding craniofacial growth particularly in the OI types III and IV, and most pronouncedly in the severe type III. In type III, the differences from population norms were the highest in patients representing the historical controls without bisphosphonate medication, but despite the fact that the biggest NT-BT intergroup differences towards normative growth among all studied types of OI were observed in type III, this group of patients even after the medication showed highest Z-score deviations from zero.

In summary, intergroup differences favouring bisphosphonate therapy were observed as more proportional growth from the skull base level to the face region, affecting both anterior–posterior and vertical dimensions of the facial bony structures, most notably through enhanced growth of the mandibular ramus. In contrast, alveolar growth—largely influenced by dentition and functional factors—showed no intergroup variation. Given that the group sizes were small and not matched for age, these findings should be interpreted with caution. A tentative conclusion is that, if bisphosphonates influence craniofacial growth in individuals with severe OI, they appear to do so in a proportional manner, though it may not reduce the occurrence of malocclusions.

### Limitations

The main limitation of our study was that the sample size of this rare disorder was limited, especially in OI types III and IV. The fact that the study group represents such a large age range, from 4 to 18 years, limits the interpretation of the findings. Despite Z-score conversion, age differences may complicate the analysis of the results, as the differences in measurements are likely to be affected by both natural growth and bisphosphonate treatment. Pubertal status was unknown, but would have been of interest since craniofacial structures also exhibit a pubertal growth spurt [44]. The study-group age-range is wide due to the retrospective nature of the study, where the radiographs had been obtained for diagnostic reasons. Obtaining excess radiographs for research purposes is generally avoided, as children and adolescents with OI undergo frequent radiographic examinations for fracture diagnostics and treatment. The pathogenic variants in the clinically diagnosed NT group were unknown, so a genetic mismatch between individuals in the NT and BT groups is possible.

The exact cumulative dose of bisphosphonate therapy was unknown on most of the individuals that had had bisphosphonate therapy, as the dosages were drawn from patient records. We did not have access to all patient records, as some of the treatment had been conducted in another hospital catchment area. Aside from calcium and vitamin D3 supplements, data about concomitant diseases or treatments were not known in either group. However, we assess this to have only little impact on the results. The information about dental agenesis, presence of dentinogenesis imperfecta or other dentin malformations were not available in this study, making it difficult to further interpret results on alveolar measurements. The method does not allow evaluation of facial structures in transversal or frontal aspect, the one that from the patient’s view probably is the most important.

## Conclusions

Bisphosphonate treatment may promote craniofacial growth in individuals with OI in vertical and sagittal dimensions. This promoting effect seemed to be slightly more pronounced in the more severe OI types, where the dimensions of the craniofacial structures differ more from the population mean norms to begin with. Understanding facial growth and eventual effects of medications on it are important in this patient group with often severe malocclusions, which in turn can complicate eating and speaking, cause tooth wear and affect a person’s appearance, thereby influencing social life and overall quality of life.

## Data Availability

All data supporting the conclusions of this study are available within the paper. The original data are not openly available due to reasons of patient confidentiality.
